# Reverse Shoulder Arthroplasty Patients Younger Than 60 Years Old Exhibit Lower Clinically Significant Single Assessment Numeric Evaluation (SANE) Scores Compared to Older Patients

**DOI:** 10.7759/cureus.46492

**Published:** 2023-10-04

**Authors:** Brendan P Stewart, Benjamin C Hawthorne, Caitlin G Dorsey, Ian J Wellington, Mark Cote, Augustus Mazzocca

**Affiliations:** 1 Department of Orthopedics, UConn Health, Farmington, USA; 2 Department of Orthopedics, UConn School of Medicine, Farmington, USA; 3 Department of Orthopaedic Surgery, Massachusetts General Hospital, Harvard Medical School, Boston, USA; 4 Department of Orthopaedic Surgery, Massachusetts General Hospital, Boston, USA

**Keywords:** clinical significance, clinical outcomes, sane scores, shoulder arthroplasty outcomes, reverse total shoulder arthroplasty

## Abstract

Introduction: The incidence of reverse shoulder arthroplasty (RTSA) in the United States has increased. Patients under 60 years old with failed rotator cuff repairs or degenerative joint disease with glenoid deformity may be candidates for RTSA and contribute to this increase. The single assessment numeric evaluation (SANE) score is a reliable post-operative scoring technique when compared with other post-operative measures. This study aimed to compare the effect of age on the likelihood of reaching clinically significant SANE scores following RTSA.

Methods: A multicenter retrospective review was performed with a consecutive series of RTSA from December 2015 to September 2021. Patients were stratified into groups based on their age at the time of operation: (1) less than 60 years old, (2) 60-69 years old, (3) 70-79 years old, and (3) greater than 80 years old. The proportions of patients in all cohorts reaching and surpassing clinically significant thresholds at each visit were determined. Likelihood ratios were determined for each age cohort to compare the likelihood of reaching clinically significant SANE scores.

Results: A total of 292 of 885 (33%) patients had completed survey data over two years and were included in the study. The 70-79-year-old group was 3.152 (p=.035) times more likely to achieve minimal clinically important difference (MCID) and 2.125 (p=.048) times more likely to achieve patient-acceptable symptomatic state (PASS) compared with patients <60 years old. The cohort who was 80+ years old was also 4.867 (p=.045) times more likely to achieve MCID compared to the <60-year-old cohort. The <60 cohort had the lowest proportion of all patient cohorts achieving MCID.

Conclusion: A lower proportion of patients younger than 60 years old undergoing RTSA achieved clinically significant post-operative SANE scores. The 70-79-year-old age group was more likely to reach MCID and PASS, and the patients who were 80+ years old were more likely to reach MCID compared to patients younger than 60 years old.

## Introduction

The incidence of reverse total shoulder arthroplasty (RTSA) has been increasing since 2012 [1]. With this increase, indications to perform RTSA have expanded, and patients under 60 years old may have failed rotator cuff repair or degenerative joint disease with glenoid deformity where RTSA may be indicated [2,3]. Overall, it has been shown that younger patients who undergo RTSA show greater improvements in post-operative measurements of range of motion (ROM), American Shoulder and Elbow Surgeons Shoulder Score (ASES), simple shoulder test (SST), and overall satisfaction and function compared to patients older than 65 [4-8].

Despite these promising results associated with specific demographics, RTSA is not without its risks. A recently published review pointed out that younger patients may experience less satisfaction in clinical outcomes following RTSA, possibly due to expectations of higher functional levels post-operatively [9]. Guy et al. described how patients had similar ROM post-operatively regardless of age; however, younger patients in their cohort had lower ASES and SST scores [3]. Additionally, it has been shown that various risk factors have predicted worse outcomes following RTSA, including opioid use prior to surgery, history of depression, proximal humerus fracture, and history of prior ipsilateral shoulder surgery [10,11].

Various markers of clinical significance can be used post-operatively. The minimal clinically important difference (MCID) is the minimal amount of change in an outcome score that the patient perceives to be important [12-15]. The substantial clinical benefit (SCB) is the amount of improvement for an outcome measure that predicts patients reporting a substantial benefit [12,15-17]. Lastly, the patient acceptable symptomatic state (PASS) is the threshold of symptoms that a patient is experiencing while still considering themselves as feeling overall well [12,14]. These clinically significant markers are determined by comparing the outcome measurements between groups that are stratified by the results of an anchor question: “Since your surgery, has there been any change in the pain in your shoulder?’’ [12,18]. By dividing the patients into cohorts based on their responses to this question, the differences between the specific outcome scores for cohorts can be compared to determine the thresholds for clinical significance. MCID is calculated by comparing patients who are identified as having no change and those who have minimal improvement on the anchor question. SCB is calculated by comparing patients who were noted to have no change and those with significant improvement. Lastly, the PASS is calculated by comparing patients with satisfactory versus unsatisfactory outcomes [12,19].

The single assessment numeric evaluation (SANE) questionnaire is a patient scoring technique that assesses improvement post-operatively by asking patients to rate the affected body part from 0% to 100%, with 100% being considered normal and the highest score [12,20-23]. The SANE score has been shown to be a reliable scoring technique when compared with other multi-question surveys [12,20-24].

In studies analyzing clinically significant improvements following RTSA, the current literature is largely focused on older cohorts. Shah et al. compared total shoulder arthroplasty (TSA) and RTSA and described a larger proportion of patients greater than 75 years old reaching SCB undergoing TSA compared to RTSA [25]. Additionally, Boettcher et al. looked at patients younger and older than 80 years old and found that all patients, no matter the age, surpassed MCID and SCB for post-operative ROM and Constant-Merley function scores [26].

This study aimed to compare cohorts of patients younger and older than 60 years old undergoing RTSA that reached MCID, SCB, and PASS for SANE scores post-operatively. We hypothesized that patients younger than 60 years old were going to be more likely to reach MCID, SCB, and PASS compared with older patients.

## Materials and methods

A multicenter retrospective review was performed on a consecutive series of RTSA from December 2015 to September 2019 via the Arthrex Arthroplasty Database (Study Number AIRR-00608-33, Arthrex Inc. Naples FL). The study was approved by the Arthrex International Grant Committee and the UConn International Review Board (IRB No. 16-042-1). Arthrex played no role in the study design, data collection, or composition of the final manuscript. Inclusion criteria included (1) RTSA performed between 2015 and 2019 for any indication and (2) completion of baseline, nine-week post-operation, 16-week post-operation, one-year post-operation, and two-year follow-up patient-reported outcomes. Patients were excluded from the study for not completing all post-operative surveys. Surveys were completed by either the operating surgeon or clinical partner within the office visit at follow-up visits at each time point. A total of 292 of 885 (33%) RTSAs performed during the study period met inclusion criteria and were, thus, included in the analysis.

To better analyze the effect of age on outcomes and the likelihood of reaching clinical significance, the patients were divided into four groups based on their age at the time of operation: (1) less than 60 years old, (2) 60-69 years old, (3) 70-79 years old, and (3) greater than 80 years old.

Surgical technique

RTSAs were performed by 15 different surgeons within the United States of America using a consistent technique (Univers ReversTM Surgical Technique; Arthrex, Naples, FL). A deltopectoral approach was used to expose the shoulder, and the biceps tendon was routinely tenodesed. The humeral head was cut at approximately 135° and then covered with a protector plate while the glenoid was prepared. Based on patient pathoanatomy and surgeon preference, a neutral, 4 mm lateralized, or 2.5 mm inferior offset baseplate was chosen with the appropriate diameter between 33 and 42 mm in 3 mm increments. The humeral canal was then broached and fitted with a standard or short press-fit humeral stem (Univers ReversTM; Arthrex, Naples, FL). Polyethylene humeral liners and spacer were used to achieve soft tissue balance. Post-operative rehabilitation was not standardized.

Statistical analysis

Metrics of clinical significance included MCID, SCB, and PASS. Clinical significance was established according to the values reported by Gowd et al: MCID is defined as an improvement in the SANE score by 29 points, SCB is defined as an increase in the SANE score by 50 points, and a PASS threshold is for SANE scores of 75 or greater [12]. These values were chosen as Gowd et al. used anchor-based questioning techniques, helping improve the reliability of these values [12,18,27]. The proportions of patients in all cohorts reaching and surpassing the clinically significant thresholds at each patient visit discussed above were determined. For the MCID and SCB, the difference between the pre- and post-operative SANE scores was used to determine if the patient crossed the threshold of 29 points and 50 points, respectively. The postoperative SANE score was used to determine whether the patient surpassed the PASS SANE score of 75.

Descriptive statistics, including mean and standard deviation (continuous variables) and the frequency and proportion (categorical variables), were determined for each age group. Differences between the age group cohorts in demographic and surgical data were determined with ANOVA, chi-square, or Fisher exact tests. Mixed effects logistic regression models were used to determine the individual effect that age and time had on achieving MCID, SCB, and PASS for SANE scores. All regression models included a random intercept to account for repeated measures on each patient. Results for the regression models are reported as odds ratios (OR) with corresponding 95% confidence intervals for the likelihood specific age group cohorts would reach clinically significant SANE scores post-operatively.

Fischer’s exact test was used in a post-hoc analysis to compare demographic information of the cohorts, including average BMI, smoking status, diabetes status, implant information, worker’s compensation, and whether the operation was a primary or revision case. A p<.05 was considered statistically significant. All statistical analyses were conducted with STATA (StataCorp 2017. Stata Statistical Software: Release 15; StataCorp LLC, College Station, TX).

## Results

A total of 292 out of 862 patients with complete data for all two-year post-operative visits were included to improve the precision of outcome measurements and, thus, decrease selection bias. Thirty-nine patients were <60 years old, 115 patients 60-69 years old, 115 patients 70-79 years old, and 23 patients older than 80 years old. Table 1 displays demographic information and implant data for the patients included in the study stratified by age cohort. The cohort of patients <60 years old was found to have a higher rate of smokers (p<.001), worker’s compensation claims (p<.001), higher rates of revision cases (p=.032), and larger glenosphere size (p=.002). There were no significant differences in BMI (p=.6792), gender (p=.101), or diabetes diagnosis (p=.908) among cohorts. Additionally, there was no significant difference between humeral inclination (p=.669), glenosphere offset (p=.715), or type of implant (p=.724) among the cohorts.

**Table 1:** Descriptive statistics of cohorts of patients undergoing RTSA. In post-hoc analysis, the <60-year-old patients were statistically more likely to have more revision cases, more likely to be smokers, and more likely to have worker’s compensation claims compared to other age cohorts. Higher proportions of patients in all age cohorts received Univers Revers implants and had a glenosphere offset 4 mm lateral and humeral inclination of 135°.

Demographic	<60 years old	60-69 years old	70-79 years old	80+ years old	Statistical significance
Number of patients	39	115	115	23	-
Mean Age (years)	55.1 ± 5.8	65.3 ± 2.6	74.1 ± 2.9	82.8 ± 2.5	-
Percent Female	59.0%	62.3%	46.5%	50.0%	p=.101
Average BMI (kg/m^2^)	31.0 ± 7.1	30.6 ± 6.1	30.3 ± 6.9	28.1 ± 3.9	p=.679
Percent Revision	15.4%	8.7%	3.5%	0%	p=.032
Current Smoker	28.2%	4.3%	5.2%	0%	p<.001
Diabetes	7.7%	12.2%	9.6%	9.5%	p=.908
Worker’s Compensation Claim	21.9%	9.6%	0.90%	0%	p<.001
Implant					p=.724
Short Stem	33.3%	39.1%	33.9%	43.5%	
Universe Reverse	66.7%	60.9%	66.1%	56.5%	
Glenosphere Size (mm)					p=.002
33	12.8%	10.4%	14.8%	26.1%	-
36	25.6%	30.4%	32.2%	26.1%	-
39	20.5%	32.2%	24.3%	39.1%	-
42	41.0%	26.1%	15.7%	0%	-
Glenosphere Offset					p=.715
4 mm Lateral	79.5%	89.6%	86.1%	87.0%	-
0 mm	17.9%	10.4%	12.2%	13.0%	-
2.5 mm Inferior	2.6%	0%	0.9%	0%	-
Humeral Inclination					p=.669
135°	100%	100%	100%	100%	-
155°	0%	0%	0%	0%	-

**Figure 1:**
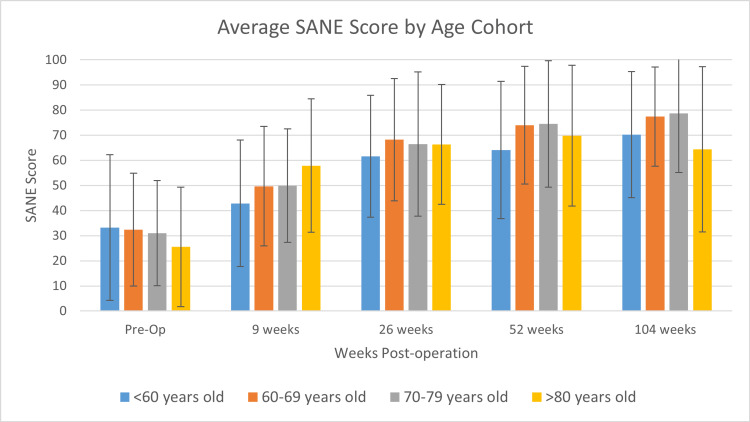
Average SANE scores for each age cohort. Patients younger than 60 years old had lower SANE scores post-operatively compared with patients in older age cohorts. Values represented as mean ± 1 standard deviation. At the 104-week visit, patients in the < 60-year-old age cohort had a significantly lower average SANE score (p=.019).

**Figure 2:**
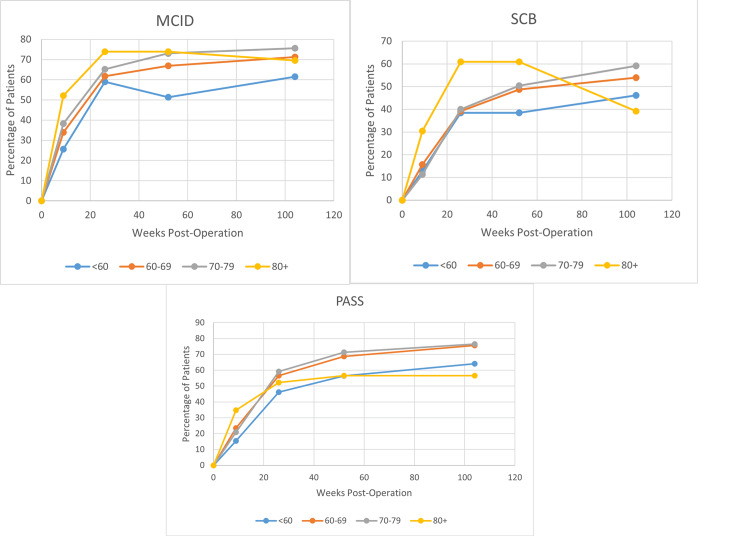
Proportions of each age cohort reaching clinically significant SANE scores. A) Proportion of each age cohort reaching MCID. The cohort of patients younger than 60 years old had the lowest proportion reaching MCID. B) The proportion of patients of each age cohort reaching SCB. The patients 80+ had the sharpest decline in the post-operative period, with 60% of patients reaching SCB after 20 weeks down to less than 40% after 100 weeks post-operation. C) The proportion of patients reaching PASS.

Figure 1 displays the average SANE scores for each age cohort at various time points throughout the study at pre-operative visits and post-operative visits at nine weeks, 26 weeks, 52 weeks (one year), and 104 weeks (two years). The <60-year-old cohort had the lowest mean SANE scores of all groups, with SANE scores of 33 at the pre-operative visit (p=.578), 43 at the 9-week post-operative visit (p=.117), 62 at the 26-week visit (p=.599), 64 at the one-year visit (p=.123), and 70 at the two-years visit (p=.019). At the two-year visit, the 80+ group's mean SANE score dropped lower than the <60-year-old cohort, with an average SANE score of 64. The largest increase in SANE scores occurred between the nine-week and 26-week visits for the < 60-year and 60-69-year-old cohorts and between pre-operative and nine-week visits for the 70-79-year-old and >80-year-old cohorts. Figure 2 displays the proportions of each age cohort reaching clinically significant SANE scores. The <60 cohort had the lowest proportion of patients achieving MCID (Figure 2A). The 80+ group has the steepest decline in patients achieving SCB over the course of the study timeframe (Figure 2B).

Table 2 displays that ORs comparing the likelihood that various age cohorts will achieve clinically significant SANE scores. The 70-79-year-old group was 3.152 (p=.035) times more likely to reach MCID and 2.125 (p=.048) times more likely to reach PASS compared to patients in the <60-year-old cohort. The cohort who was 80+ years old was also 4.867 (p=.045) times more likely to reach MCID compared with the <60-year-old cohort. There were no statistically significant differences among the various age cohorts for their likelihood of achieving SCB at the two-year mark.

**Table 2:** Odds ratios comparing age cohorts reaching clinically significant SANE scores. Odds ratios comparing each age cohort on reaching MCID, SCB, and PASS. The 70-79-year-old age cohort was 3.152 times and 2.125 times as likely to reach MCID and PASS compared to patients younger than 60 years old, respectively. The 80+ cohort was 4.867 times as likely to reach MCID as patients younger than 60 years old. Statistically significant ratios are bolded.

Comparison Group A	Comparison Group B	Odds Ratio MCID	Odds Ratio SCB	Odds Ratio PASS
<60 years old	60-69 years old	2.132	1.598	2.005
<60 years old	70-79 years old	3.152 (p=.035)	1.753	2.125 (p=.048)
<60 years old	80+ years old	4.867 (p=.045)	3.502	1.383
60-69 years old	70-79 years old	1.478	1.097	1.06
60-69 years old	80+ years old	2.283	2.191	0.69
70-79 years old	80+ years old	1.544	1.997	0.651

## Discussion

This study found that a lower percentage of patients <60 years old reached clinically significant SANE scores after undergoing RTSA at the two-year mark post-operatively. The 70-79-year-old cohort was more likely to reach MCID and PASS, and the cohort who were 80+ years old was more likely to reach MCID when compared to patients <60 years old. More specifically, patients younger than 60 years old had the lowest percentage of patients reaching MCID and were less likely than the cohorts who were 70-79 and 80+ years old to reach MCID or PASS. All age cohorts experienced the largest increase in SANE scores prior to the 26-week visit. Surprisingly, the 80+ cohort had the sharpest decline in the post-operative percentage of patients achieving SCB, with a nearly 20% decline from year one to year two.

There have been various studies that have attempted to look at clinical outcomes for RTSA based on the patient’s age. Numerous groups have described improvements in ROM, ASES, SST, and satisfaction in patients younger than 65 [4-8,28]. In contrast, Guy et al. found that patients younger than 65 years old had lower outcome scores [3]. Relatedly, Matthews et al. found younger patients to have lower ratings of functionality both pre- and post-operation [29]. Despite all of our age cohorts showing increased SANE scores between pre-operative visits and post-operative visits regardless of age, our patients <60 years old had the lowest average SANE scores at the end of the study.

The timeline of increasing SANE scores varied slightly between age groups. Patients <60 years old experienced the largest increase in average SANE scores from 43 to 62 points between the nine-week and 26-week visit. The 60-69-year-old cohort experienced an increase from 50 to 68 points between the nine-week and 26-week visit. The 70-79-year-old cohort experienced its largest increase improving from an average SANE score of 31-50 points between the pre-operative and nine-week visits. Lastly, patients older than 80 years old had the largest increase in mean SANE score from 26 to 58 points between the pre-operative and nine-week visits. Despite the largest increase occurring at slightly different times in our study, each cohort is in agreement with Grubhofer et al. and Shields et al., who both argue that a large majority of healing from shoulder arthroplasty occurs within the first six months following the operation and nearly all improvement occurs within 12 months post-operatively [30,31].

Additionally, the above findings agree with Clark et al., who described how RTSA can be safe in patients older than 80 and how age should not necessarily be a contraindication to the procedure [32]. Lastly, it should be noted that patients older than 80 exhibited a sharp decline in the percentage of patients reaching SCB at the two-year post-operative mark. As Hann et al. reported, older patient cohorts experienced a decreased range of motion following RTSA when controlling for gender, which is one possible explanation for this finding in our analysis [33].

In our post-hoc analysis, patients <60 years old were found to have a higher rate of smokers. As Hartline et al. describe, patients who identify as current smokers exhibit higher pain scores following RTSA [34]. This higher rate of smokers in the younger than 60 age cohort could be a confounding variable for the lower SANE scores and lower proportion of this cohort reaching clinical significance compared with older cohorts. This cohort also had a significantly higher percentage of worker’s compensation claims. Morris et al. showed how patients involved in worker’s compensation claims report worse outcomes post-operatively compared with patients without worker’s compensation claims [35].

Additionally, the younger than 60-year-old cohort had a significantly higher rate of revision cases. Tashjian et al. found that patients undergoing revision RTSA have an increase in pain post-operatively, which could translate to lower SANE scores seen in this group of patients within our less than 60 cohort [36]. It should be noted that the patients in our cohort identified as having a revision RTSA were patients who failed other surgeries or total shoulder arthroplasty. Further, three of these patients underwent revision RTSA for a previously failed RTSA; two patients in the 60-69-year-old age group and one patient in the 70-79-year-old group. Lastly, this cohort also had the highest percentage of patients with a glenosphere size of 42 cm, which is in contrast to literature that has shown that smaller glenosphere sizes lead to increased revision rates and worse outcomes [37,38].

Our cohorts did not exhibit statistically significant differences in average BMI. Reid et al. found that obese and non-obese patients are able to achieve clinically significant outcomes following both RTSA and TSA, which corresponds with our patient cohorts [39]. Further, despite some studies arguing that males have better postoperative outcomes following RTSA, our non-significant gender breakdown among the age cohorts is in agreement with Okohora et al. who found that RTSA outcomes are not affected by gender [40-42]. Lastly, there was no significant difference among our patients for having a diagnosis of diabetes, which is in agreement with Alsubheen et al. who point out that both diabetic and non-diabetic patients can reach clinically significant outcomes following shoulder arthroplasty [43].

One limitation of our study is that there were a large number of patients not included in this analysis due to noncompliance with study protocols and missing study data. This included patients who did not complete the required post-operative visits and surveys at all post-operative visits within the two-year mark. The 33% follow-up rate increases the chances of statistical errors, such as selection bias, and limits generalizability. Another limitation of this study is that surveys were only included through the two-year post-operative visit. As seen in our data, the patients who are 80+ years old displayed the sharpest decline in the proportion of patients achieving SCB; however, it is unknown if further declines in other age cohorts will occur beyond two years. Further research is required to determine how clinically significant SANE scores vary in post-operative visits beyond the two-year mark.

## Conclusions

In conclusion, a lower proportion of patients younger than 60 years old undergoing RTSA achieved markers of clinical significance for post-operative SANE scores. The 70-79-year-old age group was more likely to reach MCID and PASS, while the patients who are 80+ year old were more likely to reach MCID compared to younger patients. Future studies are warranted to determine whether there are differences among age cohorts in achieving clinically significant SANE scores beyond the two-year post-operative time point.
